# Cathodal HD-tDCS above the left dorsolateral prefrontal cortex increases environmentally sustainable decision-making

**DOI:** 10.3389/fnhum.2024.1395426

**Published:** 2024-06-13

**Authors:** Annika M. Wyss, Thomas Baumgartner, Emmanuel Guizar Rosales, Alexander Soutschek, Daria Knoch

**Affiliations:** ^1^Department of Social Neuroscience and Social Psychology, University of Bern, Bern, Switzerland; ^2^Department of Psychology, Ludwig Maximilian University Munich, Munich, Germany

**Keywords:** high-definition transcranial current stimulation, sustainable behavior, decision conflict, self-control, prefrontal cortex

## Abstract

Environmental sustainability is characterized by a conflict between short-term self-interest and longer-term collective interests. Self-control capacity has been proposed to be a crucial determinant of people’s ability to overcome this conflict. Yet, causal evidence is lacking, and previous research is dominated by the use of self-report measures. Here, we modulated self-control capacity by applying inhibitory high-definition transcranial current stimulation (HD-tDCS) above the left dorsolateral prefrontal cortex (dlPFC) while participants engaged in an environmentally consequential decision-making task. The task includes conflicting and low conflicting trade-offs between short-term personal interests and long-term environmental benefits. Contrary to our preregistered expectation, inhibitory HD-tDCS above the left dlPFC, presumably by reducing self-control capacity, led to more, and not less, pro-environmental behavior in conflicting decisions. We speculate that in our exceptionally environmentally friendly sample, deviating from an environmentally sustainable default required self-control capacity, and that inhibiting the left dlPFC might have reduced participants’ ability to do so.

## 1 Introduction

Many of humanity’s most pressing problems manifest as social dilemmas, which refer to situations where short-term self-interest are in conflict with longer-term collective interests. Among these, a prominent concern is environmental sustainability, specifically the excessive emission of greenhouse gases contributing to problematic climate change. Addressing this issue has led politicians to advocate for lifestyle changes toward greater environmental sustainability, which has proven to be a complex endeavor. Scientists have therefore called for more insights from the social and behavioral sciences (e.g., Creutzig et al., 2022), with a special emphasis on the cognitive (e.g., Nielsen, 2017) and neuroscientific (Doell et al., 2021; Eyring et al., 2021; Sawe and Chawla, 2021) foundations of environmentally sustainable decision-making. Following these calls, studies have increasingly focused on the role of self-control capacity, which was found to be a crucial variable underlying peoples’ (in)ability to act on their pro-environmental attitudes (Langenbach et al., 2020; Gómez-Olmedo et al., 2021; Wyss et al., 2022; Kukowski et al., 2023). To date, however, causal evidence for the role of self-control in environmentally sustainable behavior is lacking. Here, we applied high-definition transcranial direct current stimulation (HD-tDCS) to experimentally modulate cortical excitability of the dorsolateral prefrontal cortex (dlPFC), a brain region known to be involved in self-control processes (e.g., Figner et al., 2010; Levasseur-Moreau and Fecteau, 2012; Berkman, 2017; Friedman and Robbins, 2022) while participants engaged in a decision-making task with actual financial and environmental consequences.

Many people are well-intentioned to behave environmentally friendly, with the goal of contributing to a sustainable and habitable world for future generations (e.g., European Commission, 2020). However, the impact of environmentally sustainable decisions often plays out over long time horizons, and “other goals, which are closer to home both in terms of distance and time, get in the way of moving from intentions to action” (Weber, 2017, p. 2). In other words, people must forego immediate, hedonic desires (e.g., taking a shorter shower or avoid traveling by car or plane) for the benefit of environmental protection, the consequences of which are often spatially distant and temporally delayed. However, people generally display a tendency to downweigh delayed rewards relative to immediate rewards, also referred to as temporal discounting (Laibson, 1997; Berns et al., 2007; Peters and Büchel, 2011). In the domain of environmental sustainability, this bias toward present rewards has in fact shown to undermine pro-environmental attitudes and inhibit environmentally sustainable action (Franzen and Vogl, 2013; Bruderer Enzler et al., 2019; Werthschulte and Löschel, 2021).

Several research has indicated that one important resource allowing people to delay gratification for later rewards is self-control capacity (e.g., Mischel et al., 1989; Metcalfe and Mischel, 1999; Berns et al., 2007; Waegeman et al., 2014), which can be defined as the ability to regulate a short-term temptation in favor of a competing, long-term goal (e.g., Duckworth et al., 2016; Milyavskaya et al., 2019). With respect to environmentally sustainable behavior, self-control may thus allow individuals to resist short-term temptations that may interfere with their environmentally sustainable goals. Recent research has indeed provided evidence that trait self-control as well as beliefs and satisfaction about one’s self-control capacity are important determinants of environmentally sustainable behavior (Wei and Yu, 2022; Wyss et al., 2022; Jankowski and Job, 2023; Kukowski et al., 2023). However, these findings are based on correlational studies that primarily rely on self-reported assessments of self-control and/or environmentally sustainable behavior, which are susceptible to common biases such as social desirability, consistency bias or recall inaccuracy (Lange and Dewitte, 2019; Lange et al., 2023). Thus, causal research is needed to provide further insights into how self-control capacity underlies environmentally sustainable decision-making.

In order to experimentally modulate self-control capacity in an objective way and free from response biases, we aim to apply non-invasive brain stimulation above a key brain area known to be involved in self-control mechanisms: the dorsolateral prefrontal cortex (dlPFC). In fact, several brain imaging studies have shown that higher neural activity (Ballard and Knutson, 2009; Hare et al., 2014; Hung et al., 2018), higher task-independent baseline activation (e.g., Schiller et al., 2014), and more gray matter volume (Bjork et al., 2009; Liu and Feng, 2017) in the dlPFC are associated with lower discounting of delayed rewards and higher levels of self-control capacity in general. Furthermore, brain stimulation studies have shown that inhibitory stimulation of the dlPFC leads to reduced, and excitatory stimulation to increased general self-control capacity (Fecteau et al., 2007a, 2007b; Pripfl et al., 2014; Falcone et al., 2016). Moreover, inhibitory, non-invasive stimulation above the dlPFC has shown to decrease choices of delayed over smaller immediate rewards (e.g., Figner et al., 2010; Shen et al., 2016) and to increase these choices when applying excitatory stimulation (e.g., Shen et al., 2016; Nejati et al., 2018). With regard to environmental sustainability, studies have shown that greater cortical thickness and higher baseline activation in the dlPFC is positively correlated with sustainable decision-making (Baumgartner et al., 2019; Guizar Rosales et al., 2022). Together, these findings suggest that while people may be motivated to behave environmentally sustainable, they may lack the cognitive resources, such as self-control capacity, to do so.

In an attempt to provide first causal evidence for the role of self-control in sustainable behavior, Langenbach et al. (2019) inhibited the right dorsolateral prefrontal cortex (dlPFC) using continuous theta burst stimulation while participants engaged in a sustainable decision-making task. Contrary to the authors’ expectation, inhibiting the right dlPFC did not affect participants’ sustainability, thereby leaving the question of causality unanswered. Importantly, however, the left dlPFC has been predominantly linked to self-control processes in intertemporal settings (e.g., Ballard and Knutson, 2009; Figner et al., 2010; Sheffer et al., 2013; Shen et al., 2016; Yang et al., 2018; Moro et al., 2023; Zhang et al., 2023), making it a suitable candidate for modulating participants’ self-control capacity in situations where people must trade-off short-term personal interests with long-term environmental benefits. In this study, we therefore aimed to inhibit the left dlPFC to investigate whether self-control capacity is causally related to environmentally sustainable decision-making. More specifically, we applied cathodal (i.e., inhibitory) HD-tDCS, a well-established non-invasive neuromodulation technique (Kuo et al., 2013; Villamar et al., 2013; Bikson et al., 2016; Tedla et al., 2023), above participants’ left dlPFC. Participants provided behavioral measures in two separate sessions, receiving stimulation above the left dlPFC in one session and stimulation above the Vertex (i.e., active control condition) in the other session. This within-subject design, where each participant serves as their control, addresses problems of individual differences in current responsiveness and environmentally sustainable behavior baseline and enables enhanced statistical power (Thair et al., 2017). To minimize potential carry-over effects, the stimulation order was counterbalanced across participants and sessions were separated by an interval of 2 weeks.

Thus far, environmentally sustainable behavior has primarily been assessed using self-reported recalls of pro-environmental behavior and responses to hypothetical situations or intentions, which are susceptible to several biases (e.g., Lange and Dewitte, 2019). Therefore, scientists have called for measuring pro-environmental behavior with real environmental consequences (e.g., Lange et al., 2023). Following this call, we assessed environmentally sustainable behavior adapting a behavioral paradigm (Berger and Wyss, 2020) involving repeated choices consisting of an environmentally harmful, but financially beneficial option A, and an environmental-friendly option B that does not lead to a payout to the decision-maker. The environmental externality attached to the decisions was realized through the purchase and retirement of carbon emission certificates through the European Union Emission Trading System (EU-ETS; see Methods for details). This environmental decision-making task thus taps into the observation that people routinely face choices in which short-term decisions lead to harmful, long-term environmental consequences. Importantly, we created two different levels of decision-conflict (i.e., conflict and low-conflict) by combining different amounts of personal benefits (monetary benefits in CHF) with different amounts of environmental consequences (kg of CO_2_ emissions, see Methods for details). As self-control capacity is required when trying to pursue long-term motives that conflict with momentary temptations, self-control should be particularly needed in choices where individuals experience an actual conflict between financial temptations and environmental consequences. On the other hand, self-control is expected to play a negligible role in decisions that are not characterized by such a conflicting trade-off. For example, studies have shown that activity in the dlPFC and the disruption thereof to be especially sensitive to intertemporal decisions with high choice conflict characterized by intermediate relative differences between sooner-smaller and later-larger rewards (e.g., Figner et al., 2010; Jimura et al., 2018). Accordingly, we expected inhibitory HD-tDCS over the left dlPFC – compared to an active control stimulation above the Vertex – to decrease environmentally sustainable behavior in conflicting trials (conditional effect). Furthermore, we expect inhibitory HD-tDCS to decrease environmentally sustainable behavior more strongly in conflicting compared to low conflicting trials (interaction effect). The procedure and hypotheses were preregistered using the open science framework (OSF).^1^ To ensure that the effects are not driven by factors that may influence environmentally sustainable decisions such as pro-environmental attitudes (e.g., Langenbach et al., 2020), dispositional self-control (e.g., Wyss et al., 2022), or belief in the efficacy of the EU-ETS (Herweg and Schmidt, 2022), we additionally controlled for potential intraindividual differences using several questionnaires [i.e., Schwartz Value Scale (SVS) by Steg and de Groot, 2012; Brief Self-control Scale (BSCS) by Tangney et al., 2004; single item measuring EU-ETS efficacy beliefs].

## 2 Materials and methods

### 2.1 Participants and sample size

We performed an *a priori* power analysis using the package *simr* (Green and MacLeod, 2016) with 2000 simulations to determine the required sample size. The power analysis was conducted for a mixed-effects logistic regression model with a random intercept for each participant. The model included environmentally sustainable choice as our binary independent variable, and stimulation type (dlPFC vs. Vertex) and conflict level (no conflict vs. conflict) as well as the interaction thereof (stimulation type x conflict level) as independent variables. As studies investigating the effect of non-invasive brain stimulation over the prefrontal cortex on intertemporal decision-making have yielded small to medium effects (e.g., Figner et al., 2010; Shen et al., 2016; Zhang et al., 2023), we expected an Odds Ratio (*OR*) of 0.58, which corresponds to a Cohen’s *d* of about −0.3 (Chen et al., 2010) for both the conditional main effects (stimulation type, conflict level) as well as the interaction effect (stimulation type x conflict level). The simulation showed that for 36 repeated measures (18 low conflict and 18 conflict trials), a sample size of 90 participants would be needed to achieve 90% power for these effects controlling for stimulation order and session (first or second; see Supplementary Figure 1, Table 1).

Anticipating a high drop-out rate due to the Covid-19 pandemic, we recruited 128 students from the University of Bern Students of psychology, economics, and social sciences were not admitted to the experiment, as they might have been familiar with similar behavioral tasks and thus behave differently from naïve subjects. Participants with a history of neurological or mental disorders were also not admitted taking part in the experiment. 33 participants had to be excluded from the analysis due to the following reasons: sickness or no-shows (*n* = 12), comprehension problems regarding the pro-environmental behavior task (*n* = 4), technical error of the stimulation (*n* = 4), and two or more electrodes were deactivated due to high resistance levels^2^ (*n* = 13). This yielded a final sample of *n* = 95.^3^

### 2.2 Materials and protocols

#### 2.2.1 Environmental decision-making task

**Figure 1:**
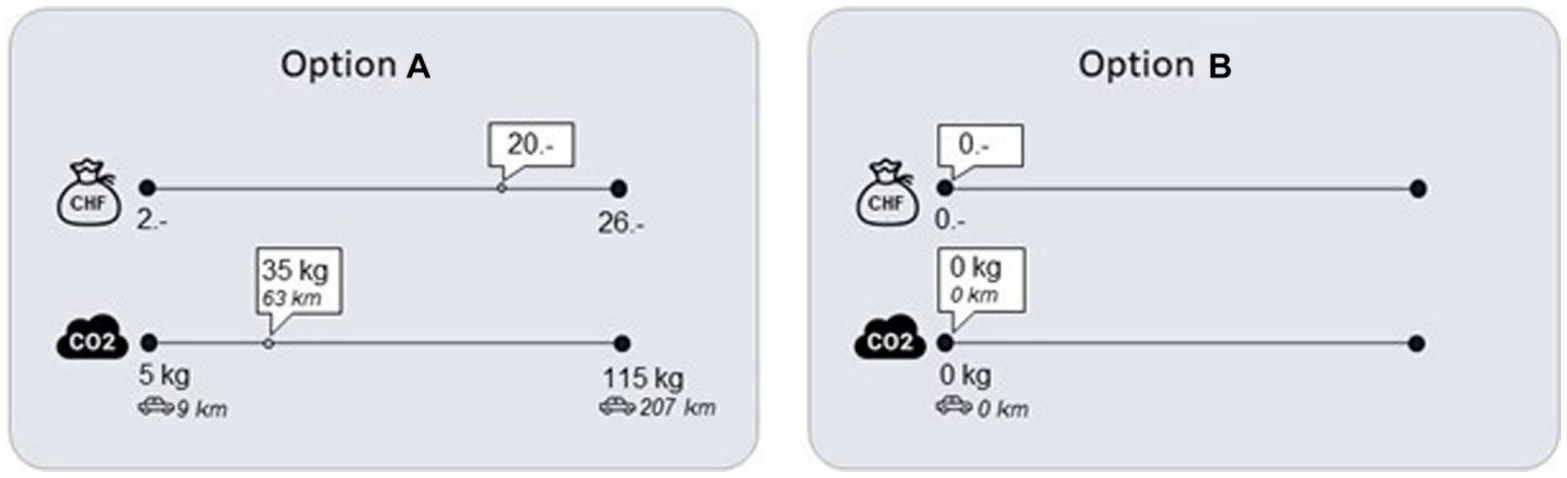
Sample decision of the environmental decision-making task (conflict trial).

To assess environmentally sustainable decision-making, we presented participants with 36 decisions about choosing an environmentally harmful but financially rewarding option A and carbon-neutral but financially non-rewarding option B (see Figure 1 for a sample decision). As previous research points to the difficulty of understanding the environmental consequence based on carbon emission estimates (Camilleri et al., 2019), the amount of carbon attached to option A was not only provided in kg of CO_2_ but also “equivalent car kilometers driven.” Participants were informed that the carbon emissions were realized using the EU-ETS, which regulates the quantity of CO_2_ emissions made by important polluters in the European Union (e.g., airlines operating within-EU flights, energy firms). Each of these polluters is endowed with tradable certificates entitling them to emit carbon. Crucially, it is possible for individuals to purchase and “retire” such certificates from the EU-ETS, with the consequence of strengthening the cap and lowering the total amount of global emissions. This method of retiring certificates is increasingly used by researchers to attach actual environmental consequences to laboratory behavior (Tavoni et al., 2011; Berger and Wyss, 2020; Ockenfels et al., 2020). After the study, we purchased these emission certificates and retired them based on participants’ decisions made in the task. The service provider for this study was the firm compensators.org.

In the task, different levels of financial rewards were paired with different amounts of carbon emissions, yielding a set of 36 distinct decisions (i.e., trials) that were randomly presented to participants. Specifically, the levels of financial rewards attached to option A were 5, 8, 11, 14, 17, 20, and 23 CHF and the amount of CO_2_ that were retired from the EU-ETS when choosing option B were 35, 50, 65, 80, 95, and 110 kg. These combinations were chosen in order to create a set of options with conflicting choices and a set of options with low conflicting choices. Although individuals are interested in both minimizing carbon emissions and maximizing financial gains, the strength of pro-environmental preferences is likely to depend on an individuals’ environmentally sustainable attitudes (see Wyss et al., 2022, for a discussion). Thus, in a student sample with high pro-environmental values, which can be expected to be the case in Switzerland (for example Bruderer Enzler and Diekmann, 2019; Hansmann et al., 2020), pairing relatively high levels of carbon emissions with relatively low levels of financial incentives is unlikely to produce an actual decision-conflict for the participants. In other words, we expected participants with high environmental concern to be unwilling to bear large environmental costs for a small financial reward, and therefore to clearly favor option B. On the other hand, we expected the pairing of relatively high financial rewards and relatively low carbon emissions to reflect choices in which participants experience an actual conflict between choosing option A and option B. Based on this assumption, we created 18 conflicting (i.e., relatively high bonus and low carbon levels) and 18 non-conflicting choices (i.e., relatively low bonus and high carbon levels; see Supplementary Table 3 for details).^4^ The trials were quasi-randomly presented to participants. More specifically, we randomly assigned 3 conflict and 3 low conflict trials to 6 blocks. The order of the blocks as well as the order of the trials within the blocks were randomly presented to participants. Reaction times as well as self-reported decision-conflict were also measured to serve as a manipulation check. Latter was assessed using the item “How easy/difficult was it for you to make the previous decision?” rated on a 6-point Likert scale from “very easy” to “very hard” after each of the 36 choices.

#### 2.2.2 Study design

**Figure 2:**
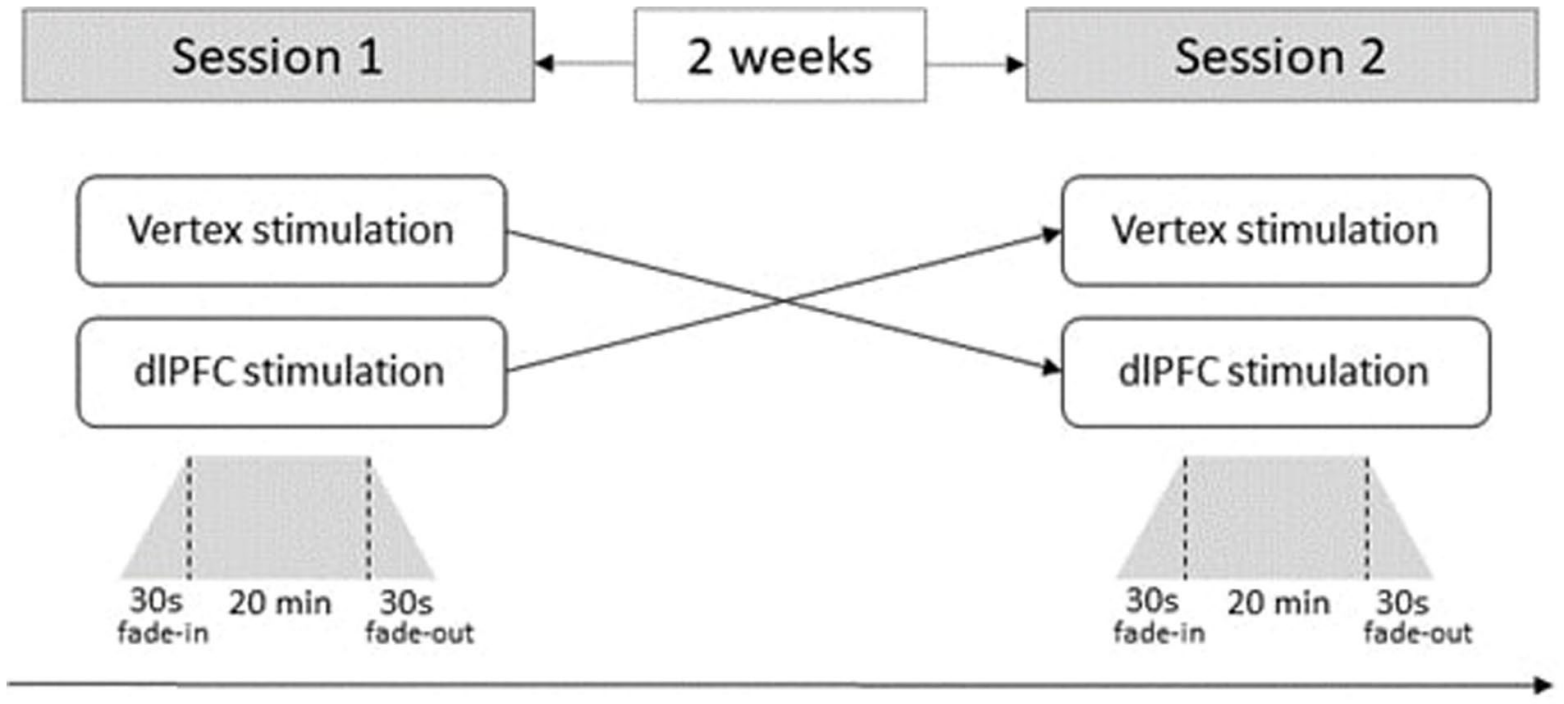
Details of the experimental procedure. The trapezia represent the electrical current of 0.5 mA delivered by each of the four return electrodes over 20 minutes with a ramp-up and ramp-down of each 30s at the beginning and the end. Order of the stimulation (Vertex vs. dlPFC) is counterbalanced.

Participants were assessed in two sessions separated by 2 weeks. For efficiency purposes, participants came to the laboratory in groups of up to four, but they did not have verbal or visual contact to each other during a session. In one session, participants received cathodal HD-tDCS over the dlPFC, and in the other session, they received cathodal HD-tDCS over the Vertex as an active control stimulation. The order of the stimulation site (dlPFC vs. Vertex stimulation) was counterbalanced across participants (see Figure 2), with 53% of all participants receiving dlPFC stimulation first. In the first session, participants read the general information about the safety of HD-tDCS and the procedure of the study and gave written consent. In both sessions, the HD-tDCS montage was installed on participants’ head using an EEG cap. Participants’ hair was removed in the corresponding regions (see below for details), and their skin was cleaned with cotton swabs soaked in alcohol. After homogeneously distributing conductive saline gel over the cleaned skin surface, the electrodes were finally fixed to the cap and additionally attached to the scalp using a rubber strap. Afterwards, participants read the instructions for the pro-environmental decision-making paradigm and, in the first session only, answered four questions tapping into their understanding of the task. Afterwards, an initial resistance check of all single electrodes was conducted and, if required, corresponding adjustments were made (i.e., adding additional gel and/or rubber bands). As soon as the HD-tDCS installation was finished, the stimulation started. Participants waited for 5 min after the onset of HD-tDCS until they were asked to start with the environmental decision-making task. After a total stimulation duration of 20 min, the stimulation stopped, and the HD-tDCS electrodes were removed from participants’ heads.

Note that in each session, one of the 36 decisions was randomly selected for payment. In the second session, participants received the decision-dependent payment from the task. After 2 weeks, participants were sent an email that included a link with online questionnaires, which they were asked to fill out. After participants had filled out the online questionnaires, they received their flat fee of CHF 50 for participating in the study.

#### 2.2.3 Application of HD-tDCS

For each participant, we used four DC stimulators plus (neuroConn, Illmenau, Germany). Each of the four return electrodes (anodes) were attached to one stimulator, and the cathode was attached to all stimulators using a connecting cable. This allowed us to monitor the resistance of each of the four anode–cathode pairs. The stimulation intensity and electrode locations were adapted from Shen et al. (2016), who demonstrated that cathodal stimulation of the left dlPFC increased participants preference for immediate rewards in an intertemporal choice task. The authors used a 4×1 ring configuration, where electrode positions corresponded roughly to C3, FT7, Fp1, and Fz, with the central electrode at F3. As the EEG cap allowed us to directly attach the electrodes to these positions, we refrained from using a ring configuration. Furthermore, we applied cathodal HD-tDCS above the Cranial Vertex as an active control with return electrodes placed on C1, FCZ, C2, and CPZ, and the central electrode on CZ. In both conditions, a current intensity of a total of 2 mA (0.5 mA per stimulator) was applied over 20 min with a ramp-up and ramp-down of each 30 s at the beginning and the end. We verified that the electrode positions and current intensity provided an electric peak field of >0.22 mA (Caulfield et al., 2022) in the left dlPFC using simulations conducted with SimNIBS (Version 3.1.0; see Supplementary material for details). The simulation showed that in the left dlPFC condition, the electric field strength in the left dlPFC was 0.289 V/m. Additionally, to ensure that in the Vertex condition the dlPFC was not stimulated, we performed a simulation for the current in the left dlPFC during Vertex stimulation, yielding an electric peak field of 0.013 V/m (see Supplementary Figures 2–5, Table 2).

Importantly, compared to relying on average resistance levels where poor resistance of single electrodes cannot be detected (Khadka et al., 2015), the use of four connected DC stimulators allowed us to continuously monitor the electrode resistance of each of the four return electrodes separately. Before the actual stimulation started, an initial resistance check was made. Electrodes with resistance levels above 150 kΩ were automatically deactivated by the system. In such cases, corresponding adjustments were made (i.e., more conductive gel and an additional rubber band). Next, we started the stimulation. All electrodes with resistance levels >150 kO were again automatically deactivated by the system. The resistance of all other electrodes (i.e., resistance <150 kO) dropped below at least 15 kΩ, and in most cases below 10 kΩ during the first 5 mins of stimulation (i.e., before the task started). 12 participants had more than one electrode deactivated in at least one of the sessions due to high resistance levels which were excluded from the analysis. In one case, an electrode was suddenly deactivated during the session as it had come loose from the holder in the EEG cap. Importantly, however, all other electrode resistance levels remained stable during the stimulation. Resistance levels prior, during, and after stimulation were monitored and documented.

#### 2.2.4 Questionnaires

In order to assess biospheric values, we used the biospheric value orientations subscale of the Schwartz Value Scale (SVS; Steg and de Groot, 2012). The four items were rated on a scale from −1 “opposed to my values” to 7 “extremely important.” Furthermore, we assessed participants’ trait self-control using the Brief Self-Control Scale (BSCS; Tangney et al., 2004). The 13 items were rated on a 5-point scale ranging from 1 “not at all like me” to 5 “very much like me.” Furthermore, participants were asked to rate a single item measuring their belief in the effectiveness of the EU-ETS (“How effective do you think the European Emissions Trading Scheme (EU-ETS) is in reducing greenhouse gases?”) on a scale from 1 “not at all effective” to 5 “very effective.”

To ensure that participants’ behavior was not influenced by potential unpleasantness caused by the stimulation, participants were asked to rate the level of unpleasantness and pain they felt during the stimulation. Pain was assessed using a scale that ranged from 0 to 10, and for each point of the scale, there was a short description available (e.g., “I feel no pain”; adapted from Hawker et al., 2011). Unpleasantness was assessed with a 7-point Likert scale ranging from 1 “very pleasant” to 7 “very unpleasant.” Both scales were administered after the environmental decision-making task during HD-tDCS.

#### 2.2.5 Data analysis

The statistical analysis of the behavioral data was performed with R (R Core Team, 2017) using the package *lme4* (Bates et al., 2015). To examine the effect of the cathodal dlPFC stimulation on environmentally sustainable behavior, we computed a mixed-effect logistic regression with dummy-coded environmentally sustainable choices (1 if yes) as dependent variable according to our *a priori* power-analysis. The model included following fixed-effects predictors: stimulation (0 = Vertex, 1 = dlPFC), conflict (0 = conflict trial, 1 = low conflict trial), the interaction term stimulation x conflict, and stimulation order (0 = dlPFC first, 1 = Vertex first), session (0 = first week, 1 = second week), as well as gender (0 = male, 1 = female) as control variables. In a next step, we additionally added pro-environmental attitudes and belief in the efficacy of the EU-ETS as control variables. In line with our preregistration protocol, we first modeled participant-specific random intercepts only. Next, we additionally included stimulation and conflict as random slopes.

## 3 Results

### 3.1 Descriptive results and manipulation check

Across both sessions and conditions, participants showed high levels of pro-environmental behavior—in 73% of the choices, participants opted for the sustainable option B (*M* = 0.73; *SD* = 0.26). Pro-environmental behavior did not differ between the first and the second session (*p* > 0.05). Mean self-reported decision-conflict across all sessions and conditions was 2.35 (*SD* = 0.68) and did not differ between the first and the second sessions (*p* > 0.05). As expected, participants were sensitive to the financial and environmental consequences attached to option A. More specifically, higher levels of financial rewards decreased pro-environmental choices (intercept-only model: *OR* = 0.12, *p* < 0.001; random-slope model: *OR* = 0.08, *p* < 0.001; see Supplementary Table 4 for more details), and higher amounts of carbon emissions increased pro-environmental choices (intercept-only model: *OR* = 10.12, *p* < 0.001, random-slope model: *OR* = 27.07, *p* < 0.001; see Supplementary Table 4). Furthermore, participants showed longer reaction times and higher levels of self-reported decision-conflict in conflict trials compared to low conflict trials (all *p* < 0.001; see Supplementary Table 5 for details), thus, participants were responsive to our two types of conflict trials (conflict vs. low conflict).

### 3.2 Regression analyses

Table 1 displays mixed-effect logistic regression results with random-intercept-only models. The dependent variable is environmentally sustainable behavior included as a dummy coded variable (0 = unsustainable choice, 1 = sustainable choice). Model 1 shows that against our hypothesis, HD-tDCS stimulation (dummy coded with 0 = Vertex stimulation and 1 = dlPFC stimulation) above the dlPFC did not decrease but increase environmentally sustainable decision-making in conflict trials (i.e., conditional effect: *OR* = 1.32; *p* = 0.005). Furthermore, the interaction effect between stimulation and conflict type (dummy coded with 0 = conflict trial and 1 = low conflict trial) did not reach statistical significance (*OR* = 0.73, *p* = 0.135), potentially due to low statistical power.^5^ Thus, cathodal HD-tDCS increased environmentally sustainable behavior at conflict trials, with no statistically significant difference between conflict and non-conflict trials.

**Table 1:** Random intercept model predicting environmentally sustainable decisions.

	Model 1			Model 2		
Predictors	*OR*	*CI*	*p*	*OR*	*CI*	*p*
Intercept	1.56	0.58–4.23	0.381	1.78	0.49–6.52	0.382
Stimulation	1.32	1.09–1.60	**0.005**	1.32	1.09–1.59	**0.005**
Conflict	98.02	68.53–140.21	**<0.001**	99.88	69.69–143.15	**<0.001**
Session	0.86	0.73–1.02	0.088	0.86	0.73–1.02	0.090
Stimulation order	0.94	0.24–3.67	0.932	0.68	0.22–2.08	0.499
Stimulation × Conflict	0.73	0.49–1.10	0.135	0.73	0.49–1.10	0.138
Gender				0.97	0.24–3.99	0.965
NEP				6.60	3.69–11.81	**<0.001**
EU-ETS efficacy belief				1.07	0.61–1.88	0.805
Random effects						
σ^2^	3.29			3.29		
τ_00_	10.94 _id_			7.19 _id_		
ICC	0.77			0.69		
N	95 _id_			95 _id_		
Observations	6,840			6,840		
Marginal R^2^ / Conditional R^2^	0.257 / 0.828			0.449 / 0.827		

Mixed-effects logistic regression model predicting environmentally sustainable choices (0 = unsustainable decision, 1 = sustainable decision). NEP refers to pro-environmental attitudes and conflict to decision-conflict. Binary variables were coded as follows: stimulation: 0 = Vertex, 1 = dlPFC; conflict: 0 = conflict trial, 1 = low conflict trial; session: 0 = week 1, 1 = week 2; stimulation order: 0 = Vertex stimulation first, 1 = dlPFC stimulation first; gender: 0 = male, 1 = female. NEP and EU-ETS efficacy beliefs were standardized.

These results remain almost identical when further controlling for gender, pro-environmental attitudes, belief in the efficacy of the EU-ETS (Model 2, conditional stimulation effect *p* = 0.005), as well as stimulation unpleasantness and trait self-control (Supplementary Table 6, conditional stimulation effect *p* = 0.007), age and major (Supplementary Table 8, conditional stimulation effect *p* = 0.005). In line with our expectation, decision-conflict in the Vertex condition (i.e., conditional effect) was a strong and significant predictor of pro-environmental decision-making in all models (*p* < 0.001), meaning that participants made more pro-environmental decisions in low conflict than in conflict trials. Furthermore, our results show that people with higher pro-environmental attitudes are more likely to opt for the environmentally friendly Option B (all models *p* < 0.001; Figure 2).

Next, we performed a mixed-effects logistic regression that included the same variables as the random-intercept-only Model (Table 1), but with random slopes for stimulation and conflict for each participant. Note that we did not perform an *a priori* power-analysis for a model with random slopes, which is likely to require more power due to the increased number of parameters. The results in Table 2 corroborate the findings from Table 1, showing a significant conditional effect of stimulation (Model 1: *OR* = 1.48, *p* = 0.043; Model 2: *OR* = 1.50, *p* = 0.038) as well as a conditional effect of conflict (both models *p* < 0.001). Additionally controlling for unpleasantness and trait self-control does not alter the results (conditional stimulation effect: *OR* = 1.54, *p* = 0.033; see Supplementary Table 7).

**Table 2:** Random intercept and random slopes model predicting environmentally sustainable decisions.

	Model 1			Model 2		
Predictors	*OR*	*CI*	*p*	*OR*	*CI*	*p*
(Intercept)	1.50	0.52–4.28	0.453	2.00	0.48–8.37	0.343
Stimulation	1.48	1.01–2.17	**0.043**	1.50	1.02–2.21	**0.038**
Conflict	172.35	73.34–405.06	**<0.001**	132.89	65.42–269.91	**<0.001**
Session	0.91	0.65–1.28	0.593	0.92	0.65–1.29	0.620
Stimulation order	0.94	0.22–4.05	0.936	0.65	0.19–2.18	0.484
Stimulation × Conflict	0.82	0.42–1.62	0.570	0.80	0.43–1.49	0.486
Gender				0.78	0.16–3.78	0.761
NEP				8.13	3.86–17.13	**<0.001**
EU-ETS efficacy beliefs				1.25	0.66–2.37	0.490
Random effects						
σ^2^	3.29			3.29		
τ_00_	11.72 _id_			8.45 _id_		
τ_11_	1.58 _id(stimulation)_			1.63 _id(stimulation)_		
	1.99 _id(conflict)_			1.84 _id(conflict)_		
ρ_01_	−0.01			0.00		
	0.01			−0.37		
ICC	0.80			0.72		
N	95 _id_			95 _id_		
Observations	6,840			6,840		
Marginal R^2^ / Conditional R^2^	0.278 / 0.857			0.459 / 0.849		

Mixed effects logistic regression model predicting environmentally sustainable choices (0 = unsustainable decision, 1 = sustainable decision). NEP refers to pro-environmental attitudes and conflict to decision-conflict. Binary variables were coded as follows: stimulation: 0 = Vertex, 1 = dlPFC; conflict: 0 = conflict trial, 1 = low conflict trial; session: 0 = week 1, 1 = week 2; stimulation order: 0 = vertex stimulation first, 1 = dlPFC stimulation first; Gender: 0 = male, 1 = female. NEP and EU-ETS efficacy beliefs were standardized.

**Figure 3:**
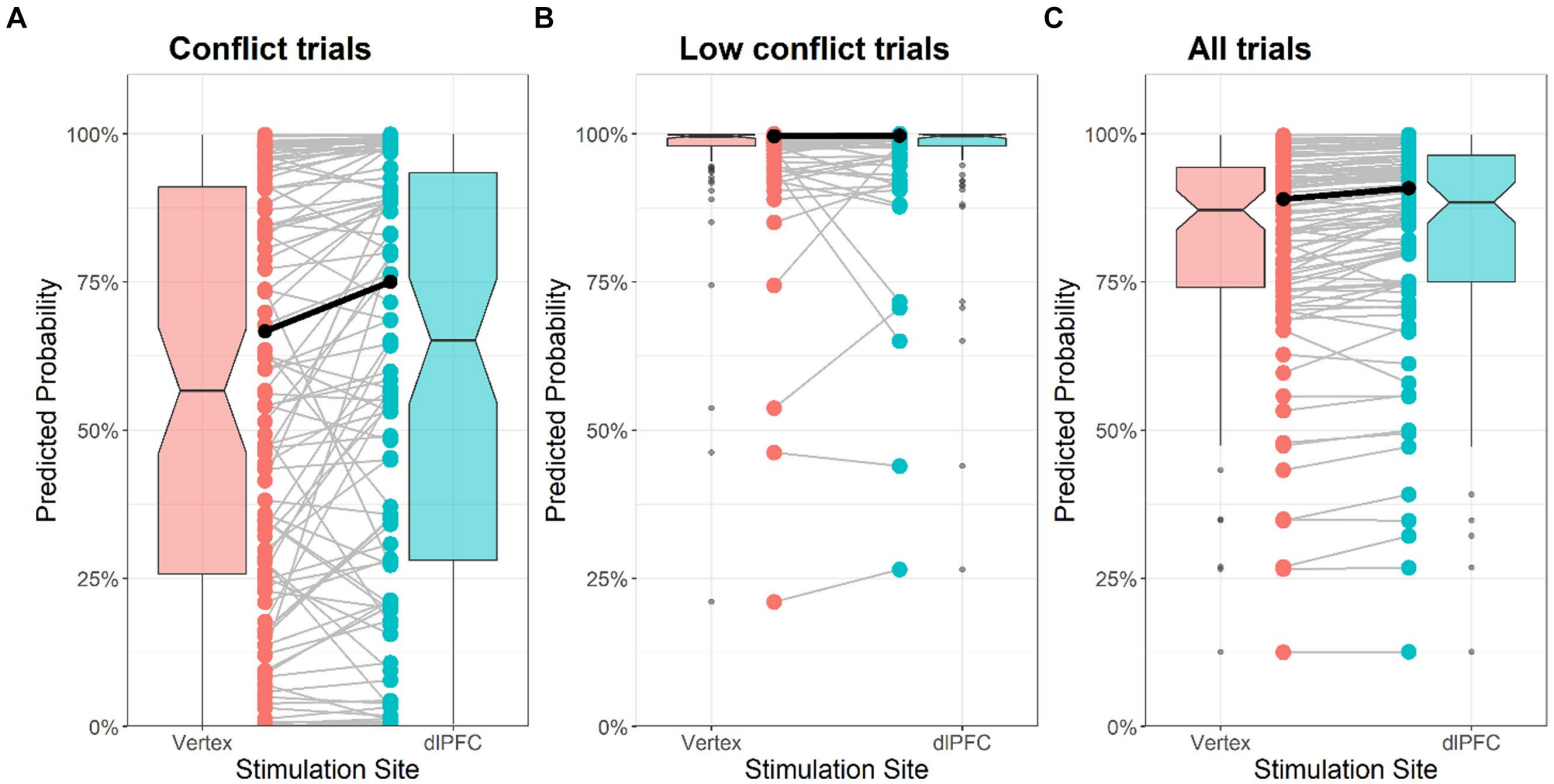
Predicted probabilities for stimulation conditions and decision-conflict. The bold lines represent the fixed effects of stimulation controlled for stimulation order, session, gender, nep, belief in EU-ETS efficacy **(A,B)** as well as conflict **(C)**. Additionally, the predicted values for each subject are shown as connected dots, as well as their distribution in form of a boxplot.

Furthermore, we visualized the predicted probabilities of per stimulation condition for pro-environmental decisions conditional on conflict trials (Figure 3A), on low conflict trials (Figure 3B) as well as controlling for decision-conflict (Figure 3C). The predicted probabilities of Figures 3A,B display the conditional stimulation effect from logistic mixed-effects regression models that include random intercepts and random slopes for stimulation and conflict for each participant controlling for session, stimulation order, gender, NEP, belief in EU-ETS efficacy. Note that the stimulation effect was statistically significant in Figure 3A (i.e., conflict trials) only (*p* = 0.038, corresponds to Model 2 in Table 2). Figure 3C displays the main effect of stimulation from a mixed-effects logistic regression model that includes random intercept and slopes for stimulation and controls for decision-conflict, session, stimulation order, gender, NEP, belief in EU-ETS efficacy (main effect of stimulation: *p* = 0.048).

### 3.3 Robustness checks

Finally, we checked whether our results on the conditional stimulation effect remain robust when excluding potential outliers. Outliers were identified based on descriptive criteria (i.e., z-score of +/− 3 of the difference in mean pro-environmental behavior between both conditions, *n* = 2) as well as based on the estimated influence of data points in the regression models (i.e., cook’s distance exceeding 4 / *n*; Nieuwenhuis et al., 2012, *n* = 3–10). In random-intercept-only models as well as models with random slopes for stimulation and conflict controlling for session and stimulation order, the conditional stimulation effect remained statistically significant (all *p*-vales <0.05, see Supplementary Tables 9–12). The effect also remained statistically significant when additionally controlling for NEP, EU-ETS efficacy beliefs. Taken together, our results suggest that—contrary to our expectation—cathodal HD-tDCS above the left dlPFC increases pro-environmental decision-making in conflicting decisions.

## 4 Discussion

In this pre-registered study, we investigated the causal relationship between self-control and environmentally sustainable decision-making by applying cathodal HD-tDCS above participants’ left dlPFC, an area known to be linked to self-control mechanisms in intertemporal choices (e.g., Ballard and Knutson, 2009; Figner et al., 2010; Shen et al., 2016; Moro et al., 2023; Zhang et al., 2023) while participants engaged in an environmental decision-making task. Contrary to our expectation, we found that inhibitory HD-tDCS did not decrease but increase environmentally sustainable behavior in conflicting decisions. In other words, in our sample, reducing the cortical excitability of the left dlPFC led to an increase in environmentally friendly behavior when a conflict between personal financial rewards and environmental consequences was present.

Our results provide evidence for previous theorizing about the role of decision conflict in environmentally sustainable behavior as a function of personal and environmental consequences and the role of self-control capacity in overcoming such conflicts (e.g., Steg and Vlek, 2009; Nielsen, 2017; Gómez-Olmedo et al., 2021; Wyss et al., 2022). Moreover, we answer the call for more consequential measures in pro-environmental behavior research, which has been dominated by the reliance on self-reports (Lange and Dewitte, 2019; Lange, 2023; Lange et al., 2023). Modulating self-control capacity by applying HD-tDCS above the dlPFC during the assessment of environmentally sustainable behavior in a task with real financial benefits and environmental harms allowed us to prevent common biases associated with self-report measures (e.g., social desirability, consistency bias, recall inaccuracy, and interpretation bias; Lange and Dewitte, 2019; Lange et al., 2023) and inflated correlations due to common method variance (Podsakoff et al., 2003) with regard to the assessment of both self-control as well as environmentally sustainable behavior.

In our view, however, our results do not imply that lower levels of self-control capacity generally lead to more environmentally sustainable behavior. In fact, research on prosocial and honest decision-making has shown that whether self-control increases or decreases prosociality or honesty depends on an individuals’ default preference, which is shaped by personality and context (Gross et al., 2018; Hackel et al., 2020; Speer et al., 2020; Wyss and Knoch, 2022; Tanaka et al., 2023). To illustrate, activity in brain areas involved in cognitive control has been found to be linked to honesty in dishonest individuals and cheating in honest individuals (Speer et al., 2020). Similarly, a recent study has shown that larger dlPFC volume is associated with more prosociality in selfish, and with less prosociality in prosocial individuals (Tanaka et al., 2023). In addition, research has provided evidence that prosocial individuals show shorter reaction times when making prosocial choices, and selfish individuals when making selfish ones (Hutcherson et al., 2015; Krajbich et al., 2015; Yamagishi et al., 2017), suggesting that higher conflict in decision-making occurs when people make choices that contradict their social preference. Thus, the dlPFC may not generally inhibit or promote prosocial and honest decision-making, but play a critical role in regulating individuals’ behavior that deviates from their default preference (Tanaka et al., 2023).

Translating these findings to the context of the present study, inhibiting the dlPFC may have led to more environmentally sustainable behavior because of the reduced self-control capacity to deviate from one’s environmentally sustainable default. Indeed, our participant sample showed very high levels of environmentally sustainable behavior with a mean proportion of environmentally friendly choices in the Vertex condition of 72% and displayed strong pro-environmental attitudes (*M* = 4.18, *SD* = 0.70, range = 1 to 7). These values are exceptionally high, as previous studies using a similar version of this task have reported an average proportion of environmentally sustainable decisions of about 39% and pro-environmental attitudes of around 3.5 (Berger and Wyss, 2020, 2021; Wyss et al., 2022). Furthermore, our participants showed shorter reaction times and reported lower levels of decision conflict when making environmentally sustainable decisions (*OR*_logrt =_ 0.85, *p* < 0.001; *OR*_conflict_ = 0.29, *p* < 0.001; see Supplementary Table 13 for more details). Thus, our data indicate that our participants may have had a default preference to act environmentally friendly, and that decisions that were consistent with this preference were easier and faster to make. Conversely, making decisions that were inconsistent with their environmentally sustainable default required more time and potentially more cognitive control. It is important to note, however, that this assumption is based on findings that are limited to the specific characteristics of our participant sample, which exhibited unusually high levels of environmentally sustainable attitudes. Future research is needed to replicate and extend these findings with more heterogeneous samples, encompassing a broader range of environmental attitudes and behaviors. Investigating individual and contextual characteristics that may influence the relationship between prefrontal self-control capacity and environmentally sustainable behavior will be crucial in advancing our understanding of sustainable decision-making.

In conclusion, inhibitory HD-tDCS above the left dlPFC, presumably by reducing self-control capacity, led to more, and not less, pro-environmental behavior in conflicting decisions. Our data suggests that our sample was exceptionally environmentally friendly and displayed a strong preference default to make environmentally friendly decisions. We therefore speculate that in our sample, deviating from this environmentally sustainable default required self-control capacity, and that inhibiting the left dlPFC might have reduced participants’ ability to do so.

## Data availability statement

The datasets presented in this study can be found in online repositories. The names of the repository/repositories and accession number(s) can be found at: https://osf.io/bh5v6/?view_only=2fafa87dd195470487d83865d5192941.

## Ethics statement

The studies involving humans were approved by Ethics Commission of the Faculty of Human Sciences (University of Bern). The studies were conducted in accordance with the local legislation and institutional requirements. The participants provided their written informed consent to participate in this study.

## Author contributions

AW: Conceptualization, Data curation, Formal analysis, Investigation, Methodology, Project administration, Writing – original draft, Writing – review & editing. TB: Conceptualization, Methodology, Writing – review & editing. ER: Conceptualization, Methodology, Writing – review & editing. AS: Conceptualization, Writing – review & editing. DK: Conceptualization, Funding acquisition, Supervision, Writing – original draft, Writing – review & editing.

## Footnotes

^1^https://osf.io/bh5v6/?view_only=2fafa87dd195470487d83865d5192941

^2^This was based on simulations of the electric peak field in the left dlPFC depending on a potential deactivation of one or more electrodes using the software SimNIBS (Version 3.1.0). The simulations showed that the failure of one electrode would still provide a sufficient electrical field strength (> 0.22 V/m; Caulfield et al., 2022; as opposed to the failure of two or more electrodes, see Supplementary Figures 2–5 for details).

^3^Please note that our initial plan, as pre-registered, was to recruit a total of *N* = 108 participants taking into account an expected dropout rate of approximately 12%. This expected drop-out rate was based on previous brain stimulation studies such as Langenbach et al. (2019) and Langenbach et al. (2022). However, due to an unexpectedly high dropout rate of participants after already the first 10 sessions due to an unexpected wave of Covid-19 and flue infections, we took the decision to recruit an additional 20 participants to ensure adequate statistical power.

^4^Data from our pilot study (*n* = 19) confirms that participants recruited from the same University reported significantly higher levels of self-reported decision-conflict in conflicting compared to non-conflicting decisions (*p* < 0.001). Importantly, participants also showed slower reaction times in conflicting decisions, and their mean level of pro-environmental decisions was significantly lower and showed more variance compared to non-conflicting decisions (all *p* < 0.001). See Supplementary Figures 6–8 for details.

^5^Note that we expected an OR of 0.58 (Cohen’s *d* = − 0.3) for the interaction effect, for which we conducted the *a priori* power analysis. This effect is much larger than the observed effect of *OR* = 0.73 (Cohen’s *d* = −0.17).
