# Supplementary material for: Cathodal HD-tDCS above the left dorsolateral prefrontal cortex increases environmentally sustainable decision-making
Source: Front Hum Neurosci. 2024 Jun 13;18:1395426. doi: 10.3389/fnhum.2024.1395426 (PMC11212476; doi:10.3389/fnhum.2024.1395426)
Supplement: Supplementary file 1 [file Data_Sheet_1.docx]

Supplementary Material

Cathodal HD-tDCS above the left dorsolateral prefrontal cortex increases environmentally sustainable decision-making

Annika M. Wyss, Thomas Baumgartner, Emmanuel Guizar Rosales, Alexander Soutschek, Daria Knoch^*^

*** Correspondence:** Daria Knoch: daria.knoch@unibe.ch

# Supplementary Tables

**Supplementary Table 1**

*A Priori Power Analysis Results*

| **N** | **Power** | **CI** |
| --- | --- | --- |
| 60 | 76.05% | 74.12, 77.91 |
| 65 | 79.70% | 77.87, 81.44 |
| 70 | 82.30% | 80.56, 83.95 |
| 75 | 84.95% | 83.31, 86.49 |
| 80 | 87.25% | 85.71, 88.68 |
| 85 | 88.50% | 87.02, 89.87 |
| **90** | **90.50%** | **89.13, 91.75** |
| 95 | 92.40% | 91.15, 93.52 |
| 100 | 93.85% | 92.71, 94.86 |

*Note.* A priori power analysis is derived from a multilevel logistic regression model that includes random intercepts for participants. Dependent variable: environmentally sustainable choices (0 = unsustainable, 1 = sustainable). Independent variables: Stimulation (0 = Vertex, 1 = dlPFC), Conflict ( 0 = conflict, 1 = low conflict), Stimulation x Conflict, Session: 0 = week 1, 1 = week 2; Stimulation order: 0 = Vertex stimulation first**.** Odds Ratio’s were set to 0.58 (Cohen’s d = -0.3) and the power analysis was conducted for the Stimulation x Conflict interaction term. 2000 simulations were conducted using the package simr (Green et al., 2016).

**Supplementary Table 2**

*Field Strength at the Left dlPFC Based on Different Stimulations*

| Stimulation | Electrode Failure | Field Strength (V/m) at left DLPFC |
| --- | --- | --- |
| left dlPFC | none | 0.2887 |
| left dlPFC | 1 | 0.2439 |
| left dlPFC | 2 | 0.1611 |
| left dlPFC | 3 | 0.1104 |
| Vertex | none | 0.0133 |

*Note*. In order to calculate the field strength (v/m) at the left dlPFC, a 5mm sphere was defined around the following MNI coordinates: x = -39, y = 37, z = 26 (see also Langenbach et al., 2019, *Scientific Reports* or Baumgartner et al., 2011, *Nature Neuroscience*). This Table displays the strength of the electrical field at the left dlPFC with cathodal HD-tDCS (-2 mA) over the left dlPFC (with 0-3 electrodes that failed) and over the vertex. Note that prior research has shown that high electric fields at the left dlPFC of 0.22–0.31 V/m result in a significantly stronger cognitive improvement than low electric fields of 0.16–0.21 V/m (Caulfield et al., 2022). Dark gray = no electrode failure, light gray = at least one electrode that failed.

**Supplementary Table 3**

*Overview of Conflict and Low-Conflict Trials Based on Carbon Emission Amounts and Bonus Levels*

|  | **5 CHF** | **8 CHF** | **11 CHF** | **14 CHF** | **17 CHF** | **20 CHF** | **23 CHF** |
| --- | --- | --- | --- | --- | --- | --- | --- |
| **20kg CO_2_**  *36km* |  |  |  |  |  |  |  |
| **35kg CO_2_**  *63 km* |  |  |  |  |  |  |  |
| **50kg CO_2_**  *90km* |  |  |  |  |  |  |  |
| **65kg CO_2_**  *117 km* |  |  |  |  |  |  |  |
| **80kg CO_2_**  *144 km* |  |  |  |  |  |  |  |
| **95kg CO_2_**  *171 km* |  |  |  |  |  |  |  |
| **110kg CO_2_**  *189 km* |  |  |  |  |  |  |  |

*Note.* Pairings of carbon and bonus levels for option A creating 18 conflicting (blue squares) and 18 low-conflicting (red squares) choices in the environmental decision-making task. Note that medium-conflicting decisions (gray) were excluded to make the distinction clearer.

**Supplementary Table 4**

*Carbon and Bonus Levels Predicting Environmentally Sustainable Decisions*

|  | Model 1 | | | | Model 2 | | |
| --- | --- | --- | --- | --- | --- | --- | --- |
| Predictors | *OR* | *CI* | *p* | *OR* | | *CI* | *p* |
| (Intercept) | 26.63 | 11.45 – 61.97 | **<0.001** | 67.90 | | 22.09 – 208.77 | **<0.001** |
| Carbon level | 10.12 | 8.67 – 11.81 | **<0.001** | 27.07 | | 16.41 – 44.63 | **<0.001** |
| Bonus level | 0.12 | 0.10 – 0.13 | **<0.001** | 0.08 | | 0.06 – 0.13 | **<0.001** |
| Random Effects | | | | | | | |
| σ^2^ | 3.29 | | | | 3.29 | | |
| τ_00_ | 16.43 _id_ | | | | 23.67 _id_ | | |
| τ_11_ |  | | | | 2.75 _id.carbon_ | | |
|  |  | | | | 1.85 _id.bonus_ | | |
| ρ_01_ |  | | | | 0.52 | | |
|  |  | | | | 0.40 | | |
| ICC | 0.83 | | | | 0.90 | | |
| N | 95 _id_ | | | | 95 _id_ | | |
| Observations | 6,840 | | | | 6,840 | | |
| Marginal R^2^ / Conditional R^2^ | 0.311 / 0.885 | | | | 0.325 / 0.929 | | |

*Note.* Carbon and bonus level are z standardized. Data includes all sessions. Model 1 includes a random intercept for each participant only. Model 2 includes random slopes for carbon and bonus levels.

**Supplementary Table 5**

*Table With Mean Reaction Time and Mean Decision Conflict Depending on Type of Trial*

|  | Low Conflict (M, SD) | Conflict (M, SD) | p (t-test) |
| --- | --- | --- | --- |
| Mean subjective decision conflict | 1.98 (1.06) | 2.74 (1.18) | *t*(1709) = 22.74, *p* < .001 |
| Mean reaction time (log) | 3.23 (2.81) | 4.71 (5.46) | *t*(1709) = 10.78, *p* < .001 |

**Supplementary Table 6**
*Random Intercept Model Predicting Environmentally Sustainable Decisions With Additional Controls*

| Predictors | *OR* | *CI* | *p* |
| --- | --- | --- | --- |
| (Intercept) | 1.77 | 0.48 – 6.49 | 0.387 |
| Stimulation | 1.32 | 1.08 – 1.62 | **0.007** |
| Conflict | 99.82 | 69.59 – 143.17 | **<0.001** |
| Session | 0.86 | 0.73 – 1.02 | 0.092 |
| Stimulation order | 0.68 | 0.22 – 2.09 | 0.502 |
| Gender | 0.97 | 0.23 – 4.01 | 0.966 |
| NEP | 6.49 | 3.26 – 12.92 | <**0.001** |
| EU-ETS efficacy belief | 1.09 | 0.62 – 1.93 | 0.768 |
| Stimulation unpleasantness | 0.99 | 0.83 – 1.18 | 0.900 |
| Self-control | 1.09 | 0.60 – 1.96 | 0.774 |
| Stimulation × Conflict | 0.74 | 0.49 – 1.11 | 0.143 |
| **Random Effects** | | | |
| σ^2^ | 3.29 | | |
| τ_00_ _id_ | 7.16 | | |
| ICC | 0.69 | | |
| N _id_ | 95 | | |
| Observations | 6,840 | | |
| Marginal R^2^ / Conditional R^2^ | 0.449 / 0.827 | | |

*Note.* Mixed-effects logistic regression model. NEP refers to pro-environmental attitudes and conflict to decision-conflict. The dependent variable was coded with 0 = unsustainable choice and 1 = sustainable choice. Binary variables were coded as follows: stimulation: 0 = Vertex, 1 = dlPFC; conflict: 0 = conflict trial, 1 = low-conflict trial; session: 0 = week 1, 1 = week 2; stimulation order: 0 = Vertex stimulation first, 1 = dlPFC stimulation first; gender: 0 = male, 1 = female. NEP, EU-ETS efficacy belief, stimulation unpleasantness, and self-control were included as standardized variables.

**Supplementary Table 7**

*Random Intercept and Random Slopes Model Predicting Environmentally Sustainable Decisions with Additional Controls*

| Predictors | *OR* | *CI* | *p* |
| --- | --- | --- | --- |
| (Intercept) | 1.95 | 0.47 – 8.18 | 0.361 |
| Stimulation | 1.54 | 1.04 – 2.30 | **0.033** |
| Conflict | 129.32 | 63.61 – 262.91 | **<0.001** |
| Session | 0.92 | 0.65 – 1.30 | 0.637 |
| Stimulation order | 0.64 | 0.19 – 2.14 | 0.469 |
| Gender | 0.80 | 0.17 – 3.86 | 0.780 |
| NEP | 7.72 | 3.63 – 16.40 | **<0.001** |
| EU-ETS efficacy belief | 1.32 | 0.68 – 2.54 | 0.412 |
| Stimulation unpleasantness | 0.93 | 0.67 – 1.29 | 0.657 |
| Self-control | 1.20 | 0.63 – 2.28 | 0.576 |
| Stimulation x Conflict | 0.81 | 0.43 – 1.50 | 0.493 |
| **Random Effects** | | | |
| σ^2^ | 3.29 | | |
| τ_00_ | 8.50_id_ | | |
| τ_11_ | 1.63 _id(stimulation)_ | | |
| τ_11_ | 1.86_id(conflict)_ | | |
| ρ_01_ | -0.01 | | |
|  | -0.41 | | |
| ICC | 0.72 | | |
| N _id_ | 95 | | |
| Observations | 6,840 | | |
| Marginal R^2^ / Conditional R^2^ | 0.461 / 0.848 | | |

*Note.* Mixed-effects logistic regression model. NEP refers to pro-environmental attitudes and conflict to decision-conflict. The dependent variable was coded with 0 = unsustainable choice and 1 = sustainable choice. Binary variables were coded as follows: stimulation: 0 = Vertex, 1 = dlPFC; conflict: 0 = conflict trial, 1 = low-conflict trial; session: 0 = week 1, 1 = week 2; stimulation order: 0 = Vertex stimulation first, 1 = dlPFC stimulation first; gender: 0 = male, 1 = female. NEP, EU-ETS efficacy belief, stimulation unpleasantness, and self-control were included as standardized variables.

**Supplementary Table 8**

*Random Intercept Model Predicting Environmentally Sustainable Decisions Additionally Controlling for Age and Major*

| *Predictors* | *Odds Ratios* | *CI* | *p* |
| --- | --- | --- | --- |
| (Intercept) | 0.00 | 0.00 – 0.55 | **0.033** |
| Stimulation | 1.32 | 1.09 – 1.60 | **0.005** |
| Conflict | 99.85 | 69.61 – 143.24 | **<0.001** |
| Session | 0.86 | 0.73 – 1.02 | 0.091 |
| Stimulation order | 0.76 | 0.26 – 2.27 | 0.625 |
| Gender | 1.45 | 0.29 – 7.27 | 0.653 |
| Age | 0.95 | 0.74 – 1.21 | 0.675 |
| Major (dummies) | INCLUDED | | |
| NEP | 13.33 | 4.65 – 38.22 | **<0.001** |
| Stimulation × Conflict | 0.73 | 0.49 – 1.11 | 0.138 |
| **Random Effects** | | | |
| σ^2^ | 3.29 | | |
| τ_00_ _id_ | 5.93 | | |
| ICC | 0.64 | | |
| N _id_ | 95 | | |
| Observations | 6840 | | |
| Marginal R^2^ / Conditional R^2^ | 0.519 / 0.828 | | |

*Note.* Mixed-effects logistic regression model. NEP refers to pro-environmental attitudes and conflict to decision-conflict. The dependent variable was coded with 0 = unsustainable choice and 1 = sustainable choice. Binary variables were coded as follows: stimulation: 0 = Vertex, 1 = dlPFC; conflict: 0 = conflict trial, 1 = low-conflict trial; session: 0 = week 1, 1 = week 2; stimulation order: 0 = Vertex stimulation first, 1 = dlPFC stimulation first; gender: 0 = male, 1 = female. The sample included participants from 15 different majors, therefore they were summarized in the table (no significant effects). NEP was included as standardized variable.

**Supplementary Table 9**

*Random Intercept Model Excluding Outliers Based on Descriptive Criteria*

|  | Model 1 | | | Model 2 | | |
| --- | --- | --- | --- | --- | --- | --- |
| Predictors | *OR* | *CI* | *p* | *OR* | *CI* | *p* |
| (Intercept) | 1.54 | 0.55 – 4.35 | 0.412 | 1.75 | 0.75 – 4.09 | 0.199 |
| Stimulation | 1.29 | 1.06 – 1.56 | **0.011** | 1.29 | 1.06 – 1.56 | **0.011** |
| Conflict | 119.14 | 80.91 – 175.44 | **<0.001** | 121.89 | 82.56 – 179.94 | **<0.001** |
| Session | 0.98 | 0.82 – 1.16 | 0.807 | 0.98 | 0.82 – 1.17 | 0.811 |
| Stimulation order | 0.91 | 0.22 – 3.73 | 0.892 | 0.64 | 0.20 – 2.05 | 0.454 |
| Stimulation × Conflict | 0.76 | 0.50 – 1.16 | 0.201 | 0.76 | 0.50 – 1.16 | 0.204 |
| NEP |  |  |  | 7.07 | 3.89 – 12.84 | **<0.001** |
| EU-ETS efficacy beliefs |  |  |  | 1.04 | 0.58 – 1.84 | 0.905 |
| Random Effects | | | | | | |
| σ^2^ | 3.29 | | | 3.29 | | |
| τ_00_ | 11.63 _id_ | | | 7.53 _id_ | | |
| ICC | 0.78 | | | 0.70 | | |
| N | 93 _id_ | | | 93 _id_ | | |
| Observations | 6,696 | | | 6,696 | | |
| Marginal R^2^ / Conditional R^2^ | 0.266 / 0.838 | | | 0.464 / 0.837 | | |

*Note.* Mixed-effects logistic regression model. NEP refers to pro-environmental attitudes and conflict to decision-conflict. The dependent variable was coded with 0 = unsustainable choice and 1 = sustainable choice. Binary variables were coded as follows: stimulation: 0 = Vertex, 1 = dlPFC; conflict: 0 = conflict trial, 1 = low-conflict trial; session: 0 = week 1, 1 = week 2; stimulation order: 0 = Vertex stimulation first, 1 = dlPFC stimulation first; gender: 0 = male, 1 = female. NEP, EU-ETS efficacy belief, stimulation unpleasantness, and self-control were included as standardized variables. For each model, outliers were defined based on the z-score of +/- 3 of the difference in mean pro-environmental behavior between both conditions (*n* = 2).

**Supplementary Table 10**

*Random Intercept Model Excluding Outliers Based on Cook’s Distance*

| *Predictors* | *OR* | *CI* | *p* |
| --- | --- | --- | --- |
| (Intercept) | 1.67 | 0.54 – 5.13 | 0.371 |
| Stimulation | 1.33 | 1.09 – 1.64 | **0.006** |
| Conflict | 132.58 | 85.74 – 205.01 | **<0.001** |
| Session | 0.89 | 0.74 – 1.07 | 0.219 |
| Stimulation order | 1.17 | 0.24 – 5.60 | 0.848 |
| Stimulation x Conflict | 0.95 | 0.58 – 1.53 | 0.822 |
| Random Effects | | | |
| σ^2^ | 3.29 | | |
| τ_00_ _id_ | 12.98 | | |
| ICC | 0.80 | | |
| N _id_ | 85 | | |
| Observations | 6,120 | | |
| Marginal R^2^ / Conditional R^2^ | 0.267 / 0.852 | | |

*Note.* Mixed-effects logistic regression model. NEP refers to pro-environmental attitudes and conflict to decision-conflict. The dependent variable was coded with 0 = unsustainable choice and 1 = sustainable choice. Binary variables were coded as follows: stimulation: 0 = Vertex, 1 = dlPFC; conflict: 0 = conflict trial, 1 = low-conflict trial; session: 0 = week 1, 1 = week 2; stimulation order: 0 = Vertex stimulation first, 1 = dlPFC stimulation first; gender: 0 = male, 1 = female. NEP, EU-ETS efficacy belief, stimulation unpleasantness, and self-control were included as standardized variables Outliers were defined based on the estimated influence of data points in the regression models (i.e., cook’s distance exceeding 4 / *n*). 10 outliers were excluded. Note that a model additionally controlling for NEP and EU-ETS efficacy beliefs did not have any outliers.

**Supplementary Table 11**

*Random Intercept and Random Slope Model Excluding Outliers Based on Descriptive Criteria*

|  | Model 1 | | | Model 2 | | |
| --- | --- | --- | --- | --- | --- | --- |
| Predictors | *OR* | *CI* | *p* | *OR* | *CI* | *p* |
| (Intercept) | 1.52 | 0.52 – 4.45 | 0.450 | 1.73 | 0.70 – 4.29 | 0.239 |
| Stimulation | 1.41 | 1.01 – 1.99 | **0.046** | 1.43 | 1.02 – 2.02 | **0.040** |
| Conflict | 186.27 | 76.03 – 456.36 | **<0.001** | 137.82 | 67.69 – 280.58 | **<0.001** |
| Session | 1.01 | 0.75 – 1.37 | 0.928 | 1.02 | 0.75 – 1.38 | 0.902 |
| Stimulation order | 0.91 | 0.21 – 4.00 | 0.898 | 0.62 | 0.18 – 2.09 | 0.436 |
| Stimulation × Conflict | 0.83 | 0.42 – 1.66 | 0.596 | 0.82 | 0.44 – 1.50 | 0.516 |
| NEP |  |  |  | 7.87 | 4.16 – 14.85 | **<0.001** |
| EU-ETS efficacy beliefs |  |  |  | 1.23 | 0.65 – 2.33 | 0.534 |
| Random Effects | | | | | | |
| σ^2^ | 3.29 | | | 3.29 | | |
| τ_00_ | 11.98 _id_ | | | 8.62 _id_ | | |
| τ_11_ | 1.04 _id(stimulation)_ | | | 1.06 _id(stimulation)_ | | |
|  | 1.94 _id(conflict)_ | | | 1.72 _id(conflict)_ | | |
| ρ_01_ | -0.03 | | | -0.02 | | |
|  | 0.03 | | | -0.41 | | |
| ICC | 0.80 | | | 0.72 | | |
| N | 93 _id_ | | | 93 _id_ | | |
| Observations | 6,696 | | | 6,696 | | |
| Marginal R^2^ / Conditional R^2^ | 0.283 / 0.859 | | | 0.471 / 0.850 | | |

*Note.* Mixed effects logistic regression model predicting environmentally sustainable choices (0 = unsustainable decision, 1 = sustainable decision). NEP refers to pro-environmental attitudes and conflict to decision-conflict. Binary variables were coded as follows: stimulation: 0 = Vertex, 1 = dlPFC; conflict: 0 = conflict trial, 1 = low conflict trial; Session: 0 = week 1, 1 = week 2; Stimulation order: 0 = Vertex stimulation first, 1 = dlPFC stimulation first; Gender: 0 = male, 1 = female. NEP and EU-ETS efficacy beliefs were standardized. For each model, outliers were defined based on the z-score of +/- 3 of the difference in mean pro-environmental behavior between both conditions (*n* = 2).

**Supplementary Table 12**

*Random Intercept and Random Slope Model Excluding Outliers Based on Cook’s Distance*

|  | Model 1 | | | Model 2 | | |
| --- | --- | --- | --- | --- | --- | --- |
| Predictors | *OR* | *CI* | *p* | *OR* | *CI* | *p* |
| (Intercept) | 1.61 | 0.56 – 4.61 | 0.377 | 1.12 | 0.35 – 3.60 | 0.852 |
| Stimulation | 1.57 | 1.07 – 2.29 | **0.020** | 1.50 | 1.01 – 2.23 | **0.046** |
| Conflict | 283.19 | 102.42 – 783.04 | **<0.001** | 104.73 | 50.55 – 216.99 | **<0.001** |
| Session | 0.86 | 0.61 – 1.21 | 0.382 | 0.95 | 0.67 – 1.35 | 0.764 |
| Stimulation Order | 0.78 | 0.17 – 3.46 | 0.740 | 0.68 | 0.25 – 1.86 | 0.457 |
| Stimulation x Conflict | 0.73 | 0.36 – 1.48 | 0.380 | 0.77 | 0.40 – 1.46 | 0.418 |
| Gender |  |  |  | 1.93 | 0.54 – 6.84 | 0.311 |
| NEP |  |  |  | 3.74 | 1.93 – 7.26 | **<0.001** |
| Belief in EU-ETS efficacy |  |  |  | 1.54 | 0.89 – 2.65 | 0.122 |
| Random Effects | | | | | | |
| σ^2^ | 3.29 | | | 3.29 | | |
| τ_00_ | 11.43 _id_ | | | 6.26 _id_ | | |
| τ_11_ | 1.47 _id(stimulation)_ | | | 1.69 _id(stimulation)_ | | |
|  | 2.26 _id(conflict)_ | | | 1.98 _id(conflict)_ | | |
| ρ_01_ | 0.02 | | | -0.03 | | |
|  | 0.34 | | | -0.61 | | |
| ICC | 0.82 | | | 0.63 | | |
| N | 92 _id_ | | | 90 _id_ | | |
| Observations | 6,624 | | | 6,480 | | |
| Marginal R^2^ / Conditional R^2^ | 0.294 / 0.872 | | | 0.445 / 0.795 | | |

*Note.* Mixed effects logistic regression model predicting environmentally sustainable choices (0 = unsustainable decision, 1 = sustainable decision). NEP refers to pro-environmental attitudes and conflict to decision-conflict. Binary variables were coded as follows: stimulation: 0 = Vertex, 1 = dlPFC; conflict: 0 = conflict trial, 1 = low conflict trial; Session: 0 = week 1, 1 = week 2; Stimulation order: 0 = Vertex stimulation first, 1 = dlPFC stimulation first; Gender: 0 = male, 1 = female. NEP and EU-ETS efficacy beliefs were standardized. For each model, outliers were defined based on the estimated influence of data points in the regression models (i.e., cook’s distance exceeding 4 / *n*). In Model 1, 3 outliers were excluded and in Model 2, 5 outliers were excluded.

**Supplementary Table 13**

*Environmentally Sustainable Default Preference Analysis*

|  | Model 1 | | | Model 2 | | |
| --- | --- | --- | --- | --- | --- | --- |
| Predictors | *OR* | *CI* | *p* | *OR* | *CI* | *p* |
| (Intercept) | 29.88 | 8.60 – 103.86 | **<0.001** | 218.42 | 49.72 – 959.63 | **<0.001** |
| Reaction time (log) | 0.85 | 0.81 – 0.88 | **<0.001** |  |  |  |
| Stimulation Order | 0.70 | 0.35 – 1.40 | 0.311 | 0.99 | 0.46 – 2.11 | 0.972 |
| Session | 0.74 | 0.65 – 0.86 | **<0.001** | 0.79 | 0.68 – 0.91 | **0.001** |
| Conflict (self-reported) |  |  |  | 0.29 | 0.24 – 0.36 | **<0.001** |
| Random Effects | | | | | | |
| σ^2^ | 3.29 | | | 3.29 | | |
| τ_00_ | 8.19 _id_ | | | 15.97 _id_ | | |
| τ_11_ | 0.03 _id(rt)_ | | | 0.67 _id(conflict)_ | | |
| ρ_01_ | -0.90 _id_ | | | -0.91 _id_ | | |
| ICC | 0.64 | | | 0.67 | | |
| N | 95 _id_ | | | 95 _id_ | | |
| Observations | 6,840 | | | 6,840 | | |
| Marginal R^2^ / Conditional R^2^ | 0.056 / 0.656 | | | 0.176 / 0.727 | | |

*Note.* Mixed effects logistic regression model predicting environmentally sustainable choices (0 = unsustainable decision, 1 = sustainable decision). Session: 0 = week 1, 1 = week 2; Stimulation order: 0 = Vertex stimulation first, 1 = dlPFC stimulation first.

# Supplementary Figures


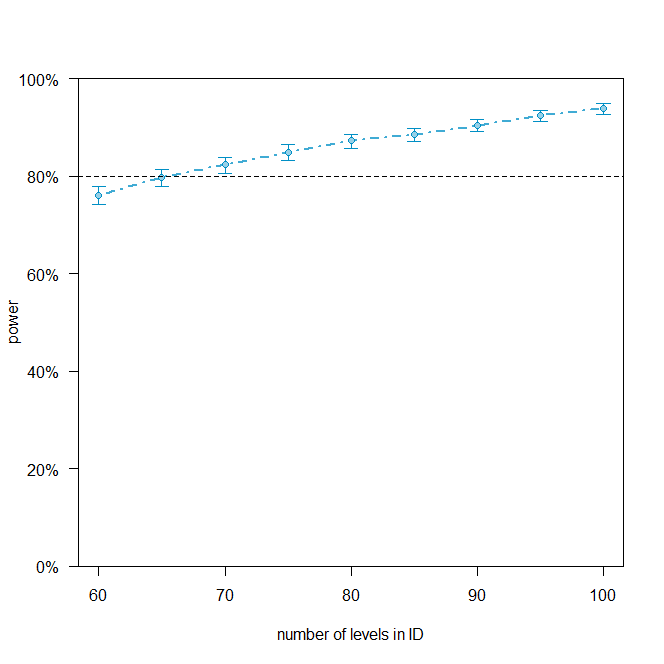
 **Supplementary Figure 1.** Graphical depiction of a priori power analysis derived from a multilevel logistic regression model that includes random intercepts for participants. Number of levels in ID = number of participants. Dependent variable: environmentally sustainable choices (0 = unsustainable, 1 = sustainable). Independent variables: Stimulation (0 = Vertex, 1 = dlPFC), Conflict ( 0 = conflict, 1 = low conflict), Stimulation x Conflict, Session: 0 = week 1, 1 = week 2; Stimulation order: 0 = Vertex stimulation first**.** Odds Ratio’s were set to 0.58 (Cohen’s d = -0.3) and the power analysis was conducted for the Stimulation x Conflict interaction term. 2000 simulations were conducted using the package simr (Green et al., 2016).


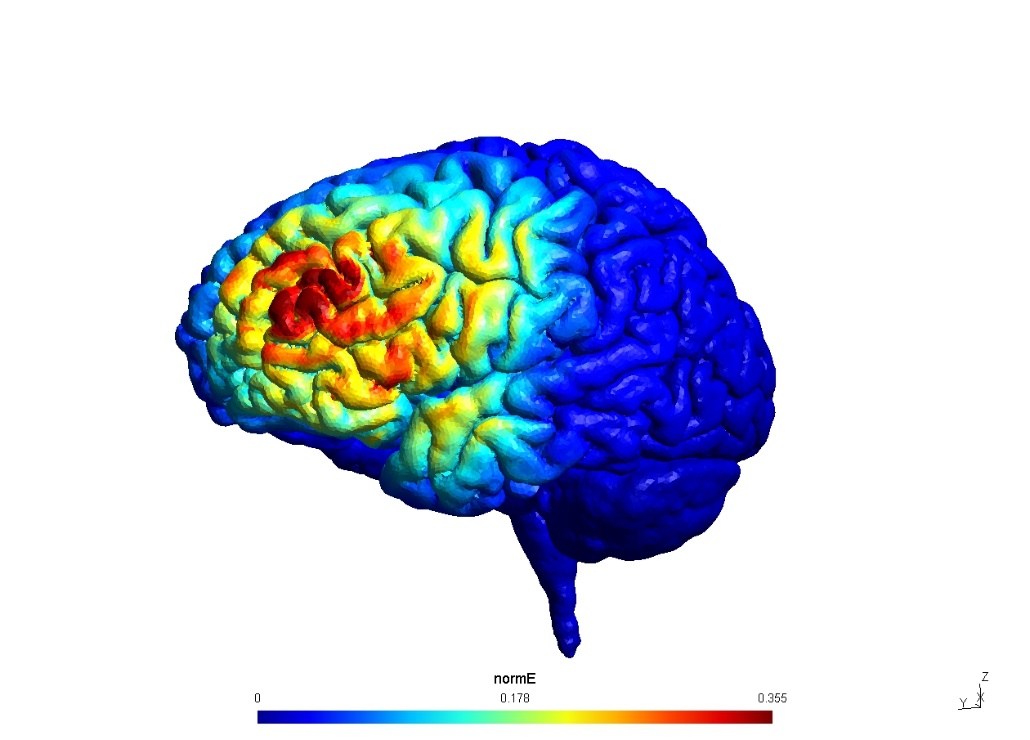


**Supplementary Figure 2.** Simulation of cathodal HD-tDCS stimulation over the left dlPFC without electrode failure based on the following electrode positions: F3 (central electrode), C3, FT7, Fp1, Fz (return electrodes). Total current of -2 mA (0.5 mA per return electrode). Thickness and diameter of electrodes were set to 2mm and 1 cm, respectively. Simulation was conducted using the software SimNIBS (Version 3.1.0).


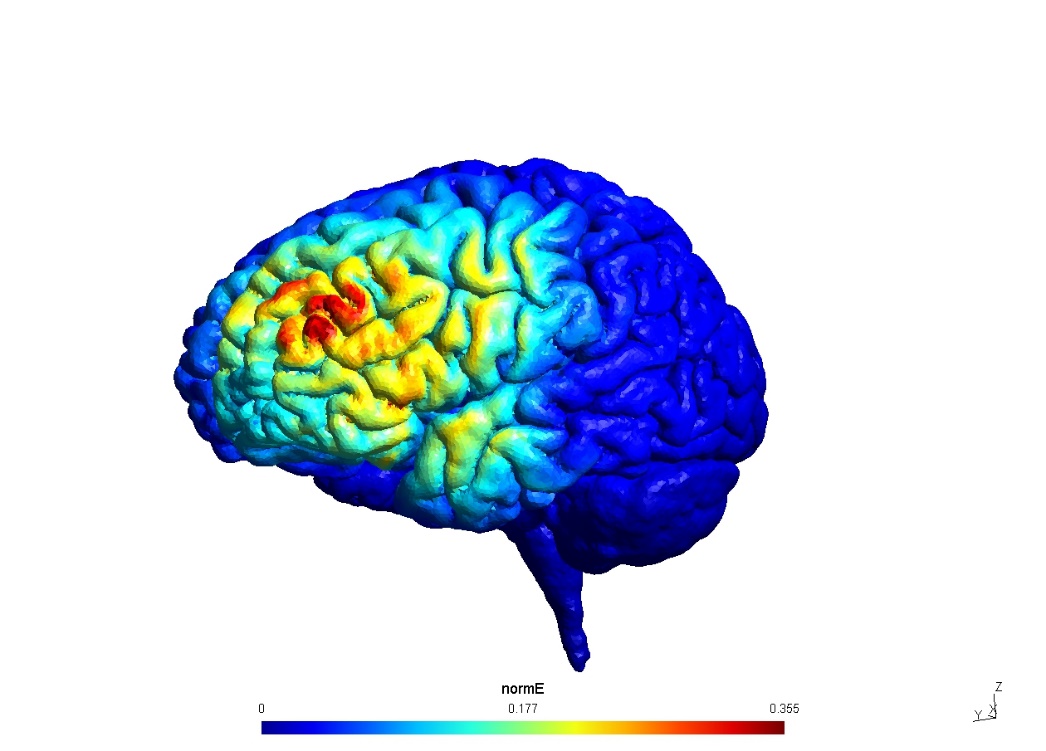


**Supplementary Figure 3.** Simulation of cathodal HD-tDCS stimulation over the left dlPFC with single electrode failure based on the following electrode positions: F3 (central electrode), C3, FT7, Fp1, Fz (return electrodes, Fp1 failed). Total current of -1.55 mA (0.5 mA on working return electrodes, 0.05 mA on Fp1 electrode). Thickness and diameter of electrodes were set to 2mm and 1 cm, respectively. Simulation was conducted using the software SimNIBS (Version 3.1.0).


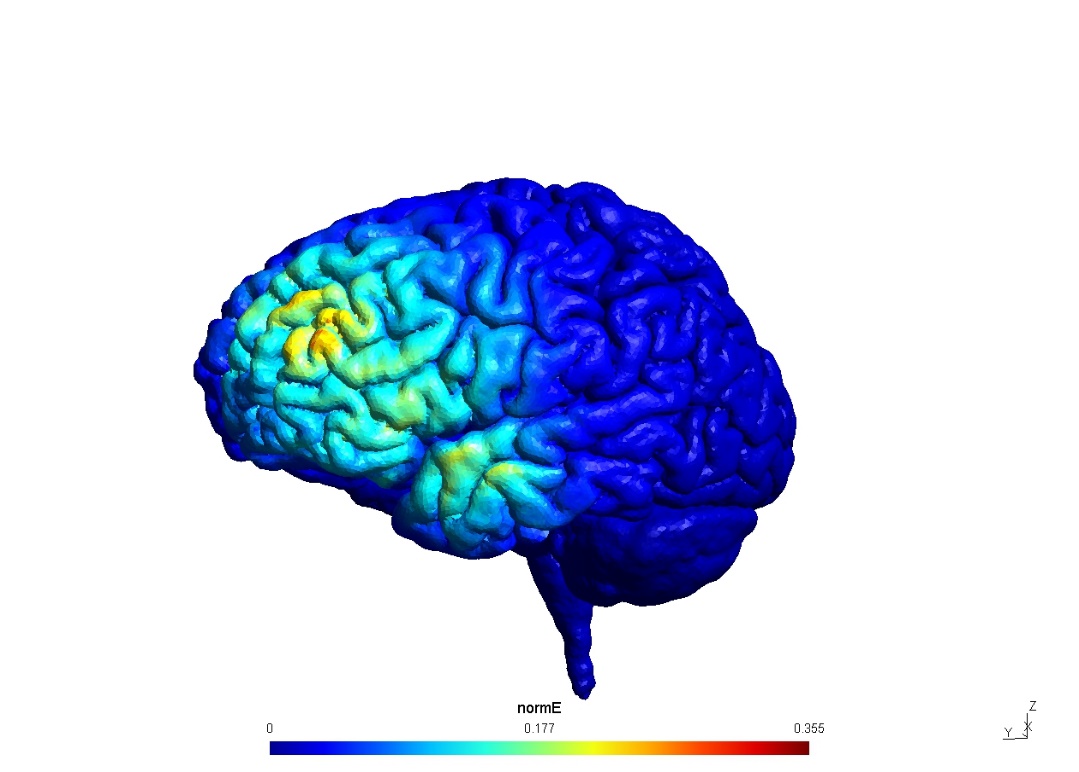


**Supplementary Figure 4.** Simulation of cathodal HD-tDCS stimulation over the left dlPFC with two electrodes failed based on the following electrode positions: F3 (central electrode), C3, FT7, Fp1, Fz (return electrodes, Fp1 and C3 failed). Total current of -1.1 mA (0.5 mA on working return electrodes, 0.05 mA on Fp1 and C3). Thickness and diameter of electrodes were set to 2mm and 1 cm, respectively. Simulation was conducted using the software SimNIBS (Version 3.1.0).


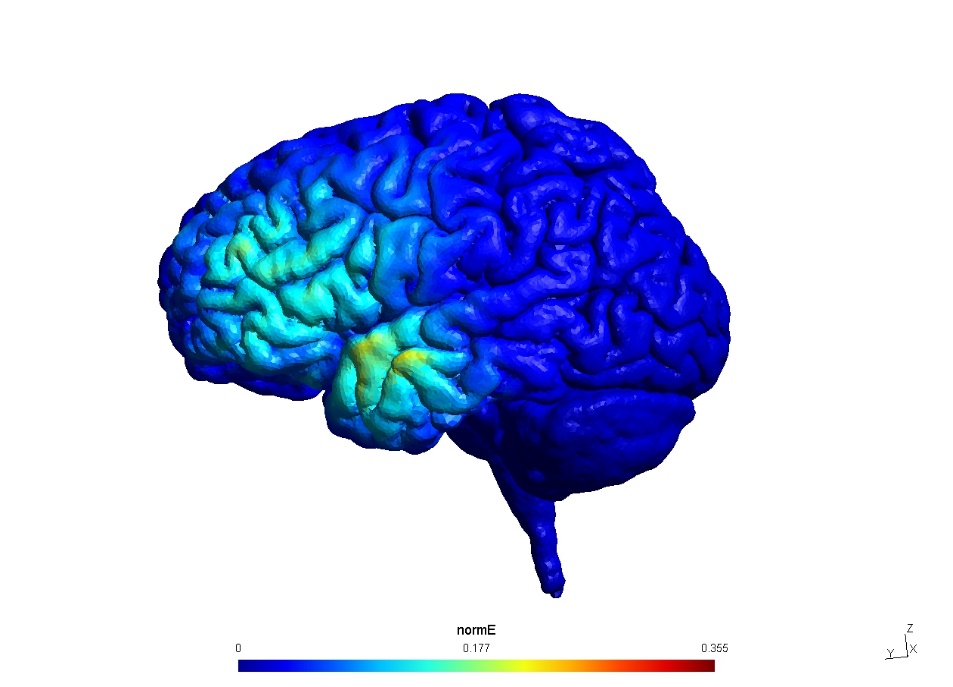


**Supplementary Figure 5.** Simulation of cathodal HD-tDCS stimulation over the left dlPFC with three electrodes failed based on the following electrode positions: F3 (central electrode), C3, FT7, Fp1, Fz (only FT7 working). Total current of -0.65 mA (0.5 mA on working return electrode, 0.05 mA on C3, Fp1, Fz). Thickness and diameter of electrodes were set to 2mm and 1 cm, respectively. Simulation was conducted using the software SimNIBS (Version 3.1.0).


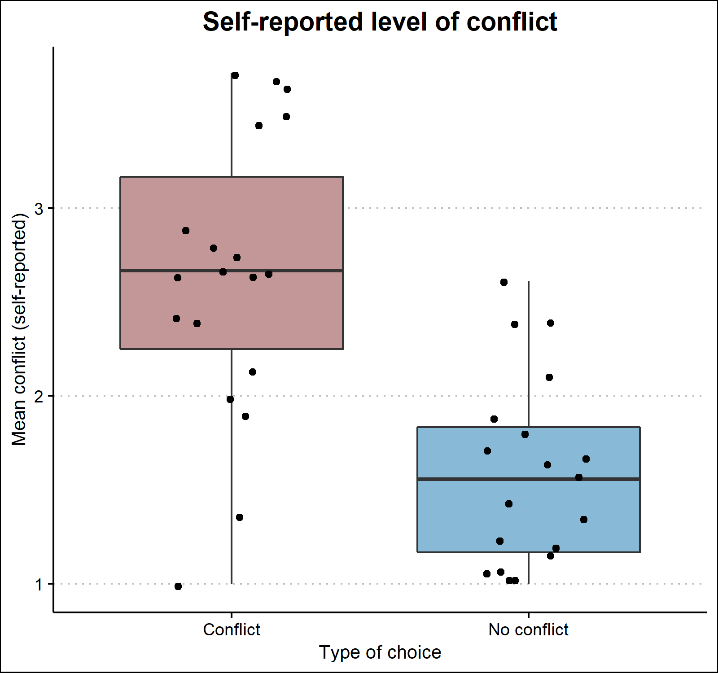
 **Supplementary Figure 6.** Data from pilot study showing self-reported level of conflict depending on type of trial. Mean conflict reflects the mean of self-reported decision-conflict of all pro-environmental decisions across all 36 choices (18 conflicting and 18 non-conflicting choices) on a scale from 1 (it was extremely easy to make the decision) to 6 (it was extremely hard to make the decision) in Week 1 (M_conflict_ = 2.64, SD_conflict_ = 0.76, M_no-conflict_ = 1.59, SD_no-conflict_ = 0.50, t(18) = 7.26, p < .001). N = 19.


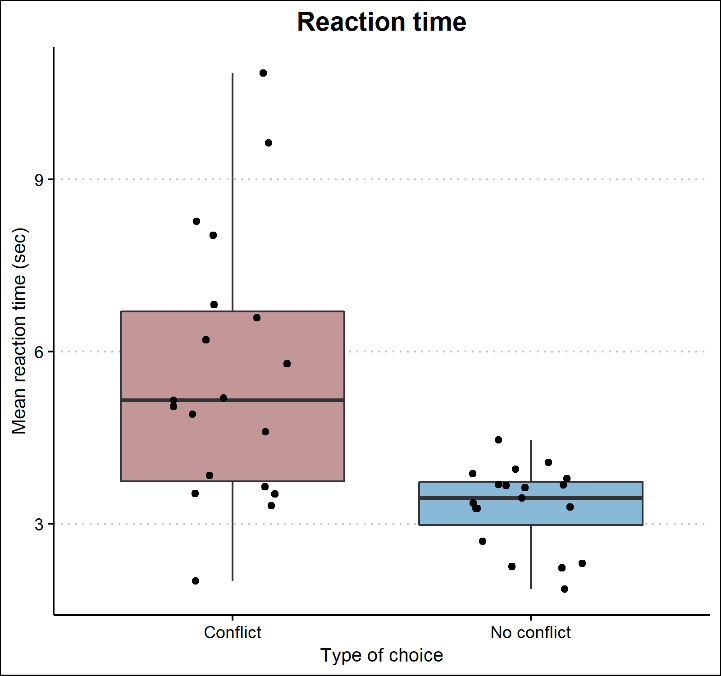


**Supplementary Figure 7.** Reaction times from pilot study depending on type of trial. Mean reaction times in seconds across all 36 choices (18 conflicting and 18 non-conflicting choices) in Week 1 (*M_conflict_* = 5.64, *SD_conflict_* = 2.31, *M_no-conflict_* = 3.30, *SD_no-conflict_* = 0.71, *t*(18) = 4.24, *p* < .001). N = 19.

**
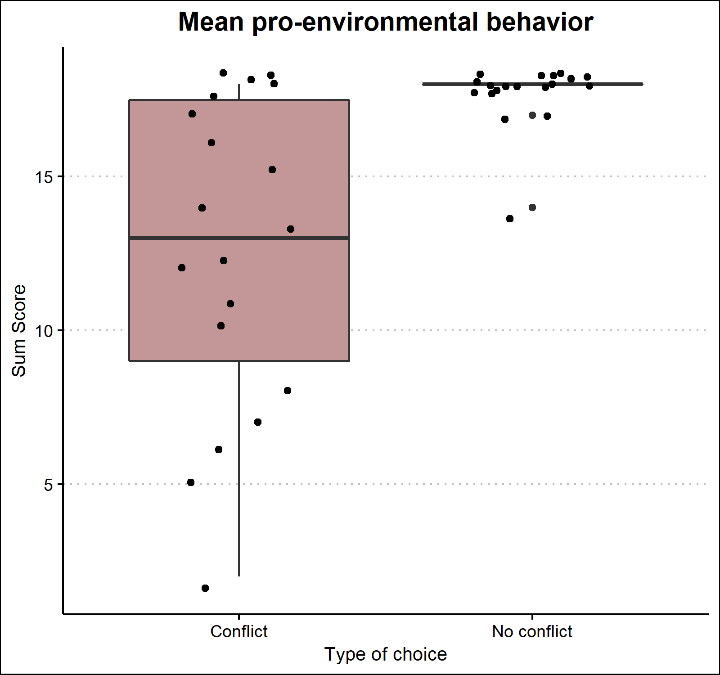
**

**Supplementary Figure 8.** Data from pilot study showing mean environmental sustainability depending on type of trial. Mean environmental sustainability across all 36 choices (18 conflicting and 18 non-conflicting choices) in Week 1 (*M_conflict_* = 12.53, *SD_conflict_* = 5.06, *M_no-conflict_* = 17.68, *SD_no-conflict_* = 0.95, *t*(18) = -4.88, *p* < .001). N = 19.
